# Polychlorinated Biphenyls Induce Mitochondrial Dysfunction in SH-SY5Y Neuroblastoma Cells

**DOI:** 10.1371/journal.pone.0129481

**Published:** 2015-06-23

**Authors:** Stefania Cocco, Agnese Secondo, Adelaide Del Viscovo, Claudio Procaccini, Luigi Formisano, Cristina Franco, Alba Esposito, Antonella Scorziello, Giuseppe Matarese, Gianfranco Di Renzo, Lorella Maria Teresa Canzoniero

**Affiliations:** 1 Division of Pharmacology, Dept. of Neuroscience and Reproductive and Odontostomatological Sciences, School of Medicine, “Federico II” University of Naples, Via Sergio Pansini 5, Naples, 80131, Italy; 2 Dept. of Science and Technology-DST, University of Sannio, via Port'Arsa 11, 82100 Benevento, Italy; 3 Laboratorio di Immunologia, Istituto di Endocrinologia e Oncologia Sperimentale, Consiglio Nazionale delle Ricerche (IEOS-CNR), c/o Dipartimento di Medicina Molecolare e Biotecnologie Mediche, Università di Napoli “Federico II”, Napoli, Italy; 4 Dipartimento di Medicina e Chirurgia, Università di Salerno, Baronissi Campus, Baronissi, Salerno, Italy; 5 IRCCS MultiMedica, Milano, Italy; The University of Iowa, UNITED STATES

## Abstract

Chronic exposure to polychlorinated biphenyls (PCBs), ubiquitous environmental contaminants, can adversely affect the development and function of the nervous system. Here we evaluated the effect of PCB exposure on mitochondrial function using the PCB mixture Aroclor-1254 (A1254) in SH-SY5Y neuroblastoma cells. A 6-hour exposure to A1254 (5 μg/ml) reduced cellular ATP production by 45%±7, and mitochondrial membrane potential, detected by TMRE, by 49%±7. Consistently, A1254 significantly decreased oxidative phosphorylation and aerobic glycolysis measured by extracellular flux analyzer. Furthermore, the activity of mitochondrial protein complexes I, II, and IV, but not V (ATPase), measured by BN-PAGE technique, was significantly reduced after 6-hour exposure to A1254. The addition of pyruvic acid during exposure to A1254 significantly prevent A1254-induced cell injury, restoring resting mitochondrial membrane potential, ATP levels, oxidative phosphorylation and aerobic glycolysis. Furthermore, pyruvic acid significantly preserved the activity of mitochondrial complexes I, II and IV and increased basal activity of complex V. Collectively, the present results indicate that the neurotoxicity of A1254 depends on the impairment of oxidative phosphorylation, aerobic glycolysis, and mitochondrial complexes I, II, and IV activity and it was counteracted by pyruvic acid.

## Introduction

Polychlorinated biphenyls (PCBs), widely used in a variety of industrial and consumer products for several decades, were banned in the 1970s. Owing to their extensive industrial use and chemical stability, PCBs are still ubiquitous and persistent environmental contaminants [1]. It has been shown that chronic exposure to PCBs can impair cognitive development and function of the nervous system [2]. Indeed, whereas in children prolonged PCB exposure can result in hyperactivity and memory deficits, in adults it may result in motor activity deficits [2]. Although clear evidence of a correlation between neurological damage and PCB exposure has been widely described, the molecular and the cellular mechanisms involved in these alterations still need clarification. What is known so far is that alterations in Ca^2+^ ions may elicit PCB-induced toxic effects. In particular, PCBs can modify Ca^2+^ homeostasis in different subcellular compartments like synaptosomes, mitochondria, and microsomes [3–6]. Further research has also indicated that PCB-induced increases in cytosolic Ca^2+^ yield nitric oxide (NO) synthesis; a case in point is that the Ca^2+^-dependent NO-guanylyl cyclase-cGMP-PKG pathway triggered by PCBs widely contributes to their toxicity [7]. Given that the increase in both cytosolic Ca^2+^ and NO can exert a toxic effect on mitochondria [8–12] we sought to evaluate the role of mitochondria in PCB-induced neurotoxic effects. For this purpose, we examined in neuroblastoma cells the effect of Aroclor-1254 (A1254), a PCB mixture, on mitochondrial function by evaluating several mitochondrial parameters including their oxidative activity, mitochondrial membrane potential, oxidative phosphorylation, and specific mitochondrial complex activity as well as aerobic glycolysis. Furthermore, we evaluated whether pyruvic acid, an energy substrate that has been shown to be neuroprotective in several experimental toxicity models [13–18] could counteract A1254-induced mitochondrial damage.

## Methods

### Cell Culture

Human SH-SY5Y cells (LGC Standards S.r.l., Sesto San Giovanni, Italy) were cultured as monolayers in polystyrene dishes in Dulbecco's modified Eagle's medium (DMEM) containing 15% heat-inactivated fetal bovine serum, 1% L-glutamine (200 mM), 1% sodium pyruvate (100 mM), 100 IU/ml penicillin, and 100 μg/ml streptomycin. All of the above reagents were purchased from Invitrogen (Milan, Italy). Cells were grown in a humidified incubator at 37°C in a 5% CO_2_ atmosphere, and the medium was changed every 2 days. Each experiment was performed using cells (passages 15–30) plated on multiwell plates. After 24 h and 48 h of cell seeding, cells were incubated with A1254 (stock solution; 1 mg/ml) in DMEM without serum.

### Determination of Cell Viability Evaluated as Mitochondrial Activity

Cell viability was evaluated using the 3[4,5-dimethylthiazol-2-y1]-2,5-diphenyltetrazolium bromide (MTT, Sigma-Aldrich, Milan, Italy) test was performed essentially as previously described [19]. In this test, the dye MTT is metabolized by viable mitochondria to a colored product that can be detected using a spectrophotometer at a wavelength of 540 nm. Data obtained from three independent experimental sessions were expressed as a percentage of the mitochondrial viability of sham-treated cultures.

### Nitric Oxide Detection with DAF-2 Assay

After the incubation with A1254, A1254+ pyruvic acid, and pyruvic acid alone, cells were loaded with 10 μM 4,5-diaminofluorescein-2-diacetate (DAF-2DA) in a humidified 5% CO_2_ atmosphere at 37°C for 30 min [20]. For control experiments, cells were incubated only in normal Krebs solution. Subsequently, fluorescent cells were fixed with 4% (w/v) paraformaldehyde in PBS for 5 min at room temperature. This procedure permits a subsequent densytometric analysis with the fluorescence microscope Nikon Eclipse E400 (Nikon, Torrance, CA) set at an excitation/emission wavelength of 495/515 nm. Fluorescent images are then stored and analyzed with Pro-Plus software (Media Cybernetics, Silver Springs, MD). The data are reported as percentage of the fluorescence of the control group.

### Determination of Mitochondrial Membrane Potential

Mitochondrial membrane potential (ΔΨ) was assessed using the fluorescent dye tetramethyl rhodamine ethyl ester (TMRE) in the ‘redistribution mode' as previously described [21]. Confocal images were obtained using a Zeiss inverted 510 confocal laser-scanning microscope and a 63X oil-immersion objective. The illumination intensity of 543 mm Xenon laser, used to excite TMRE fluorescence, was kept to a minimum (0.5%) of laser output to avoid phototoxicity.

### Quantification of ATP Content

ATP content was measured by a commercial bioluminescent assay (ATP bioluminescent assay kit, Sigma, St Louis, Missouri, USA) according to the manufacturer’s instruction. Briefly, ATP was extracted by boiling the samples in a solution containing 100 mM TRIS, 4 mM EDTA, pH 7.75. After centrifugation at 10 000 X g for 60 s, samples were diluted at 1:50 in dilution buffer (Sigma, FL-AA). To obtain bioluminescence measurements with a standard luminometer, 100 μl of supernatant was mixed with 100μl of luciferin-luciferase solution. The standard curve of ATP was obtained by serial dilution of 2 μM ATP solution [22].

### Blue Native Page and Histochemical Staining

Blue-native (BN) PAGE and subsequent in-gel enzymatic colorimetric reactions were performed essentially as previously described [23] but with minor modifications. After isolation of mitochondria and solubilization of individual respiratory chain complexes with 10% dodecyl maltoside, 30μg of each sample was loaded on a 6–13% gradient acrylamide gel and subjected to electrophoresis. After runs, for each set of gels, one gel was fixed and stained with Coomassie Blue G to quantify total amount of each complex and normalize the corresponding in gel activity and the other was used to determine enzymatic activities by colorimetric reactions. Complex I (NADH-Dehydrogenase) activity was determined by incubating the gel with 0.1 M Tris–HCl, 768 mM glycine, 0.1 mM β-NADH, and 0.04% Nitro blue tetrazolium (NTB), at pH 7.4 and RT. As an index of complex II activity, succinate dehydrogenase (SDH) activity was evaluated by incubating the gel in 0.1M Tris–HCl, 100 mM glycine, 10 mM succinic acid, and 1 mg/ml NTB at pH 7.4 and RT. Complex IV (COX) activity was estimated by incubating BN-PAGE gels with 5 mg 3,3’–diaminobenzidine tetrahydrochloride (DAB) dissolved in 9 ml phosphate buffer (0.05 M pH 7.4), 1 ml catalase (20 μg/ml), 10 mg cytocrome C, and 750 mg sucrose. The original color of the reactions of complex I, II or IV was preserved by fixing the gels with 50% methanol and 10% acetic acid. Complex V activity was obtained by incubation of BN-PAGE gel in 35 mM Tris, 270 mM Glycine, 14 mM MgSO4, 0.2% Pb(NO3)2, and 8 mM ATP at pH 7.8 and RT. The reaction was stopped by addition of 50% methanol. Finally, the gels were washed in distilled water. The original color of complex I and complex II bands (violet), of complex IV (red), and complex V (white) were analyzed as a grey scale image by means of Bio-Rad Imaging Densitometer (GS-800, Quantity One, BioRad). Band intensities were expressed as absolute values in arbitrary units. The optical density (OD) of each band was plotted against the OD values derived from the Coomassie Blue gel.

### Metabolism Assays

Real-time measurements of oxygen consumption rate (OCR) and extracellular acidification rate (ECAR) were made using an XF-96 Extracellular Flux Analyzer (Seahorse Bioscience). Cells were plated in XF-96 plates (Seahorse Bioscience) at the concentration of 20000 cells/well. OCR was measured in XF media (non-buffered DMEM medium, containing 10 mM glucose, 2 mM L-glutamine, and 1 mM sodium pyruvate), under basal conditions and in response to 5 μM oligomycin, 1.5 μM of carbonylcyanide-4- (trifluoromethoxy)-phenylhydrazone (FCCP) and 1 μM of Antimycin and Rotenone (all from Sigma Aldrich). ECAR was measured in XF media in basal condition and in response to 10 mM glucose, 5 μM oligomycin and 100 mM of 2-Deoxy-D-glucose (2-DG). Data are expressed as mean ± SEM from 3 separate experiments. n = 5 replicated per sample. Two-tailed Mann–Whitney test was used for statistical analysis.

### Drugs and Chemicals

All the chemicals were of analytical grade and were purchased from Sigma (Milan, Italy). A 1254 (lot No. LB15060) had a 99% purity. All chemicals were diluted in cell culture medium, and, for those requiring dilution in DMSO, the final DMSO concentration was 1%. DMSO was added to control cells at the same concentrations as those used in treated cells. DMSO by itself did not cause any cell toxicity.

### Statistical Analysis

Experiments were repeated at least three times, and data were analyzed by one-way ANOVA, followed by Newman-Keuls test.

## Results

### Effect of Aroclor-1254 (A1254) on cell survival, ATP production and mitochondrial membrane potential (mΔΨ) in SH-SY5Y cells in SH-SY5Y cells

**Fig 1:**
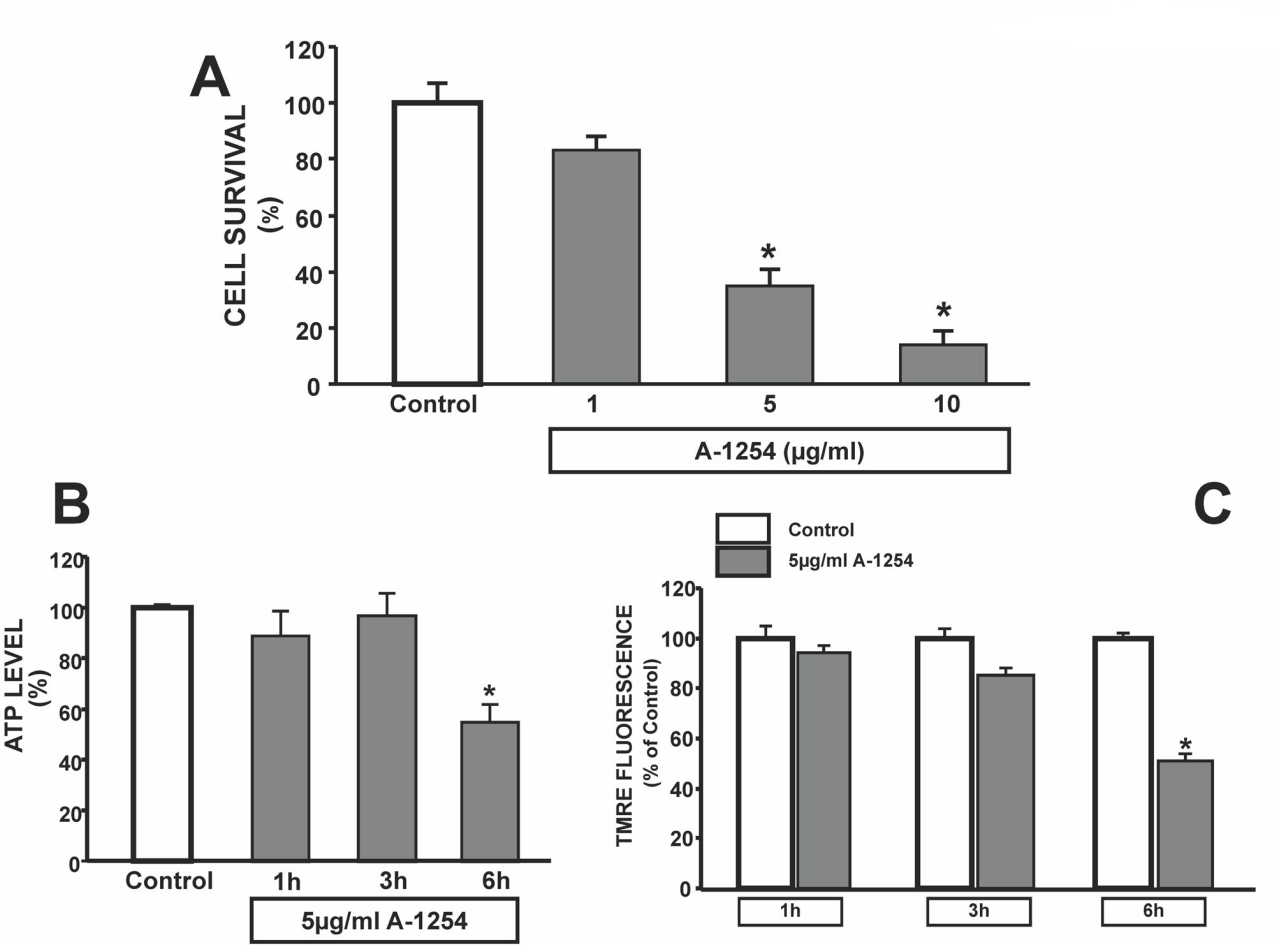
Effect of A1254 on mitochondrial oxidative activity, on ATP production and mitochondrial membrane potential after 24hr exposure in SH-SY5Y cells. Panel A. Cell Viability in SH-SY5Y cells exposed (24 hrs) to increasing concentrations of A1254 (A1254; 1–5–10 μg/ml). Each bar represents the mean ± SEM (10 different experiments) of cell viability assessed by MTT assay. *P < 0.05 vs control group Panel B. Effect of 5 μg/ml A1254 on ATP content in SH-SY5Y cells. The bar graph represents the ATP content measured after 1, 3 and 6 hrs exposure to PCB. ATP levels were normalized to protein content of each sample. Each bar represents the mean (±S.E.M.) obtained from 6 different sessions in three independent experimental sessions. *P < 0.05 vs control group. Panel C. TMRE analysis of SH-SY5Y cells exposed to A-1254 (5 μg/ml) for 1, 3 and 6 hrs. Cumulative data are expressed as mean ± S.E.M. of TMRE fluorescence changes (percentage of control values taken as 100%). The intensity of fluorescence was calculated as arbitrary units of the relative fluorescence intensities of each sample with MetaMorph software analysis, and expressed as percentage of normoxic values. *P<0.05 versus all.

To examine the role played by PCBs on cell survival measured as mitochondrial activity, we exposed SH-SY5Y neuroblastoma cells to increasing concentrations of A1254 (1, 5 and 10 μg/ml). A dose-related reduction in mitochondrial activity, measured by MTT analysis, occurred after 24 hrs of exposure (Fig 1A). The concentration of 5 μg/ml A1254, which significantly damaged approximately 60% of cells after 24 hrs incubation (Fig 1A), was chosen to carry out our experiments. To study the triggering events leading to cell death, ATP production and mitochondrial membrane potential variation were measured at early times of exposure to A1254. After 6 hours of exposure, A1254 did not change cell survival rate (data not shown) but induced a reduction in ATP production of 45%±7, whereas no significant difference were observed after 1 and 3 hour exposure (Fig 1B). The decline in ATP levels was accompanied by a reduction in mΔΨ, which was examined with TMRE, a fluorescent probe sensitive to the variation of this potential (Fig 1C). Indeed, A1254 induced a reduction in mitochondrial membrane potential of 49%±7 after 6 hours of exposure, whereas no significant variation was found at 1 and 3 hours after exposure (Fig 1C).

### Effect of pyruvic acid on cell survival and NO levels in SH-SY5Y cells exposed to Aroclor-1254 (A1254)

**Fig 2:**
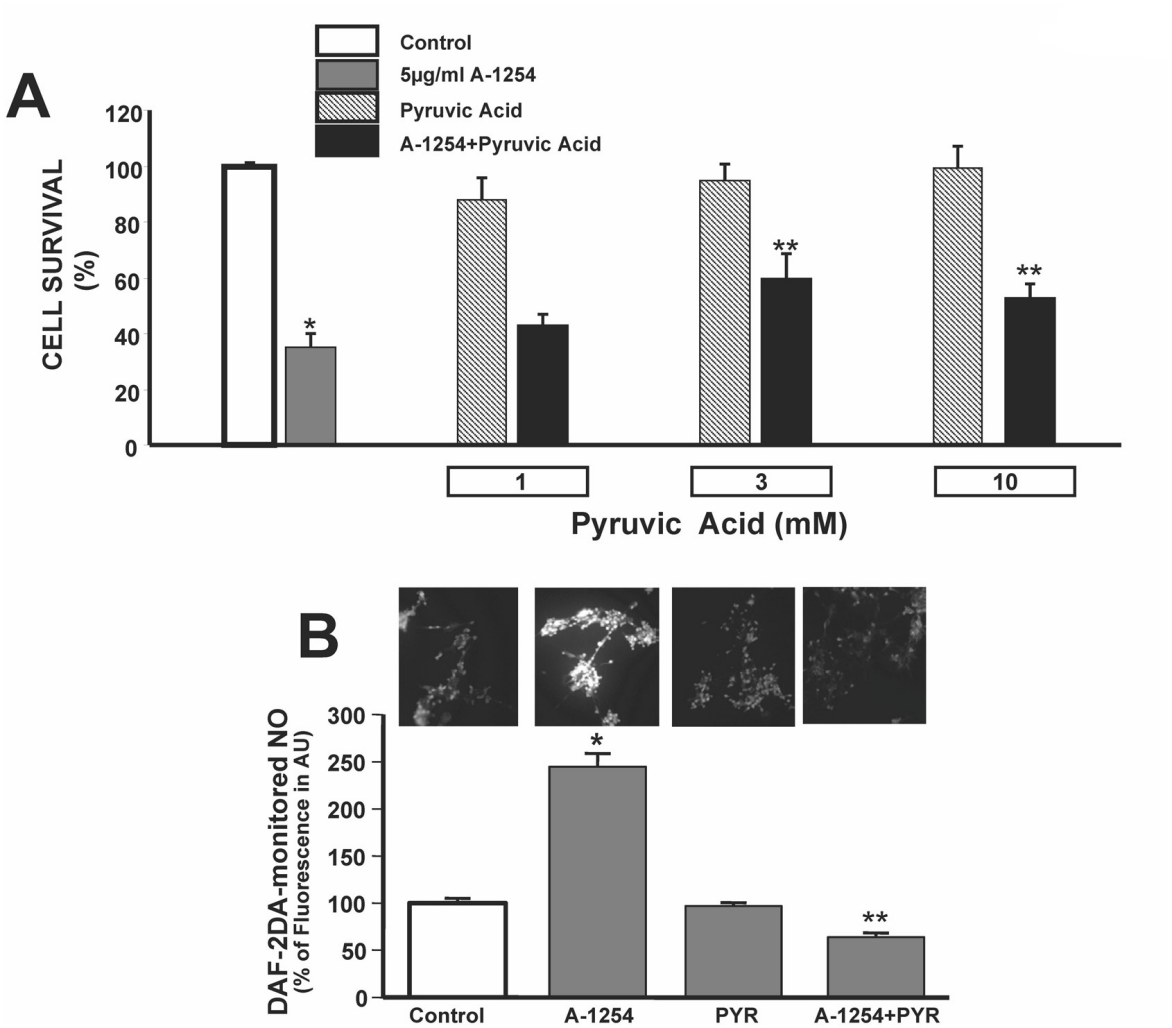
Effect of Pyruvic Acid on A1254-Induced Cell Injury and DAF-2DA-monitored NO in SH-SY5Y cells. Panel A. Quantification of cell viability assessed by MTT assay in SH-SY5Y cells exposed to A1254 (5 μg/ml/24 hrs) in the presence of different concentrations of pyruvic acid (1-3-10 mM). Each bar represents the mean of 10 separate experiments. *P < 0.05 versus Control, **P < 0.05 versus A1254. Panel B. Representative fluorescent images of neuroblastoma cells stained with DAF-2DA probe in control conditions, after 6 hr exposure to A1254 (5 μg/ml), in the presence of pyruvic acid (10 mM) alone and after 6 hr exposure to A1254+ pyruvic acid (10 mM). The intensity of fluorescence was calculated as arbitrary units of the relative fluorescence intensities of each sample, and expressed as percentage of control values (considered as 100%). *P<0.05 versus control cells; **P < 0.05 versus A1254.

Considering the effect of A1254 on ATP levels and mΔΨ, we evaluated whether pyruvic acid, an energy substrate that has been shown to be neuroprotective in several experimental toxicity models, could prevent A1254-induced cell death in neuronal cells. Pyruvic acid (1, 3 and 10 mM) was added to the incubation medium of SH-SY5Y cells during A1254 exposure. Interestingly, pyruvic acid improved the survival of SHSY5Ycells while being exposed to 24 hours of A1254 (5 μg/ml) (Fig 2A). Interestingly, pyruvic acid (10 mM) prevented A-1254-induced increase of intracellular nitric oxide monitored by DAF-2DA, thus suggesting that pyruvic acid may inhibit endogenous reactive oxygen species production (Fig 2B).

### Effect of pyruvic acid on Aroclor-1254-induced decrease of mΔΨ and ATP levels in SH-SY5Y cells

**Fig 3:**
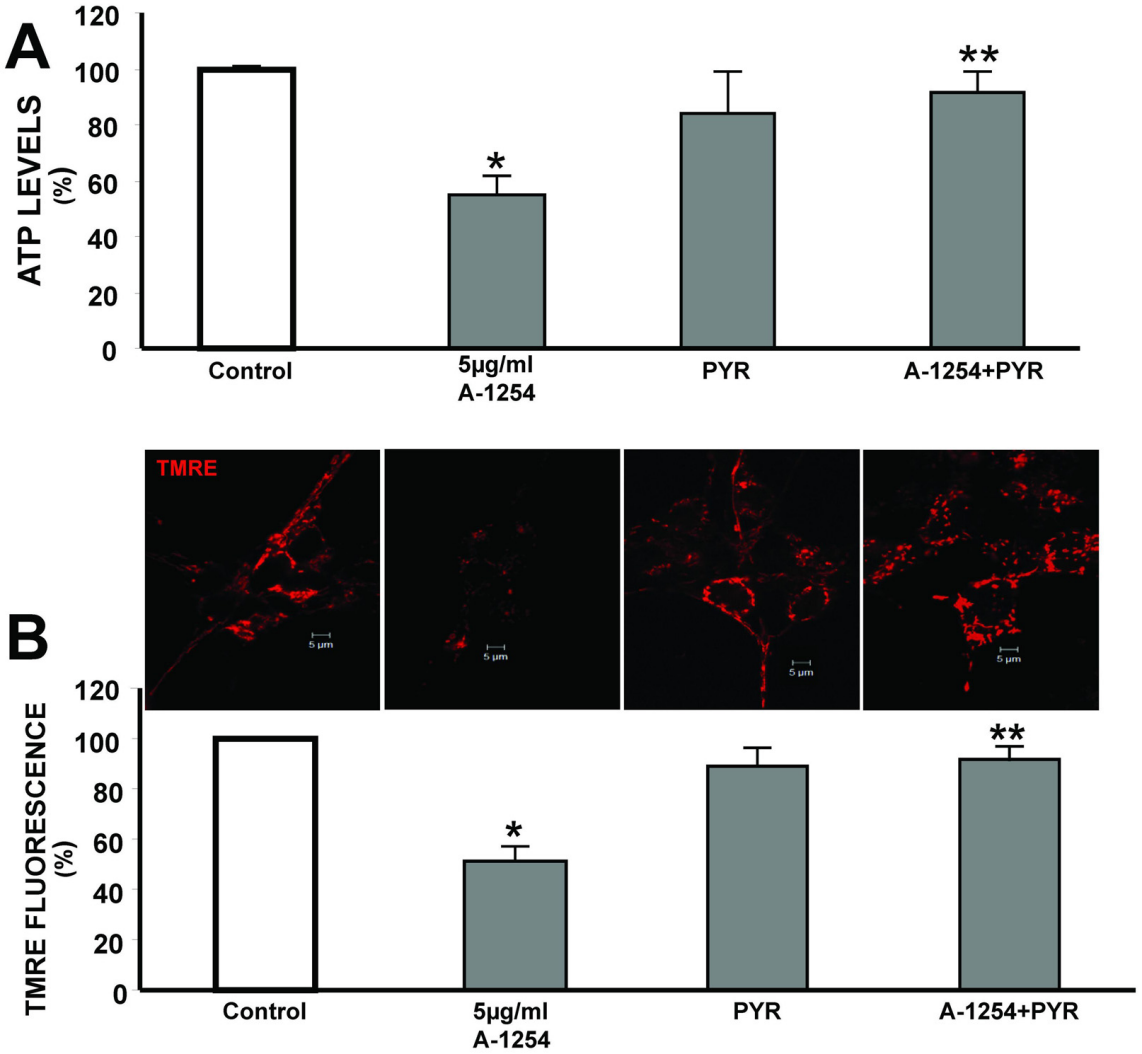
Effect of pyruvic acid on ATP production and A1254-induced decrease of membrane mitochondrial potential (mΔΨ) in SH-SY5Y cells. Panel A. Quantification of ATP content in SH-SY5Y cells exposed to A1254 (5 μg/ml) ± 3 mM pyruvic acid for 6hrs. Each bar represents the mean (±S.E.M.) of 6 different experimental values in three independent experimental sessions.*P < 0.05 vs control cells, P<0.05 versus A1254.Panel B. Top. Confocal images of neuroblastoma cells stained with TMRE in control cells and after 6 hr treatment with PCB mixture A1254 in the presence or absence of 3 mM pyruvic acid. Bottom. Cumulative data are expressed as mean ± S.E.M. of TMRE fluorescence changes (percentage of control values taken as 100%). The intensity of fluorescence was evaluated in single cells with MetaMorph software analysis. *P<0.05 versus control cells (CTL), **P<0.05 versus A1254. Effect of A1254 (5 μg/ml) on mitochondrial membrane potential in SH-SY5Y cells. A) Confocal images of neuroblastoma cells stained with TMRE probe in control cells and after 6 hr treatment with PCB mixture A1254. B) TMRE analysis of SH-SY5Y cells exposed to PCB mixture. Cumulative data are expressed as the mean of changes in TMRE fluorescence obtained in 7 different experiments. These values are expressed as percentages of control values. The intensity of fluorescence was evaluated in single cells by means of Meta Morph software analysis. *P<0.05 versus control cells (DMSO exposed).

Three millimolar of pyruvate, a lowest effective concentration able to ameliorate mitochondrial activity, prevented A1254-induced reduction of ATP levels by 21%, compared with cells treated with 5 μg/ml A1254 (Fig 3A). Similarly, at the same concentration, it prevented A1254-induced reduction of mΔΨ by 36.8%, compared with cells exposed to 5 μg/ml A1254 (Fig 3B).

### Effect of pyruvic acid on Aroclor-1254-induced decrease of oxidative phosphorylation and aerobic glycolysis in SH-SY5Y cells

**Fig 4:**
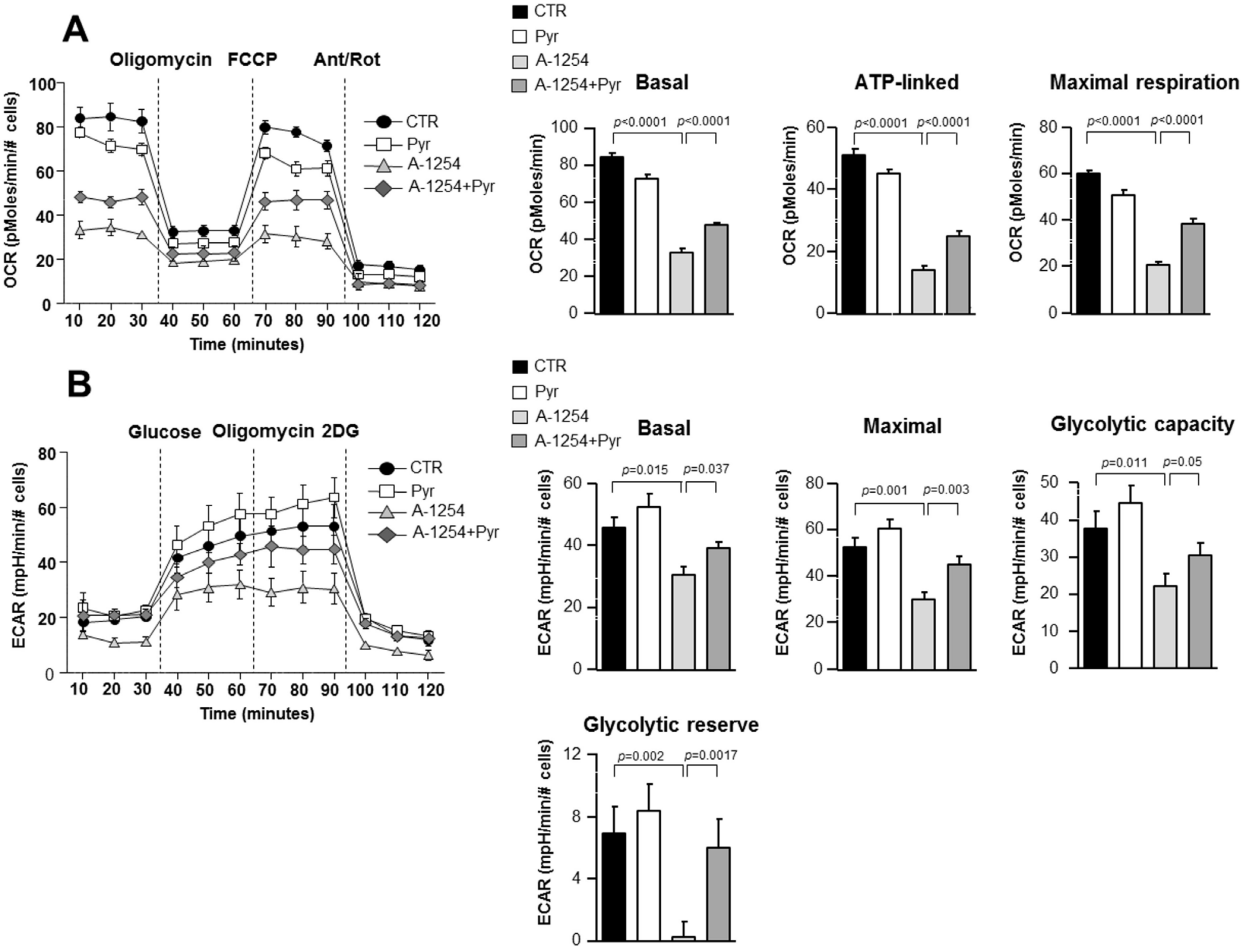
Resilience of mitochondrial function in response to pyruvate after A1254 exposure in SH-SY5Y cells. Panel A. OCR measurement after 6 hrs exposure to A1254 (5 μg/ml) in the presence or absence of pyruvic acid. Basal OCR, ATP-linked and maximal respiration were measured by the injection of oligomycin (5 μM), FCCP (1.5 μM) and antimycin (1 μM) plus rotenone (1 μM) at indicated times. Panel B. ECAR measurement after 6 hrs exposure to A1254 (5 μg/ml) in the presence or absence of pyruvic acid. Basal ECAR, maximal ECAR, glycolitic capacity and glycolytic reserve were measured by injection of glucose (10 mM), oligomycin (5 μM) and 2-DG (100 mM) at the time indicated. Data = mean±SE. Data were normalized to number of cells.

To test whether A1254 could affect cellular metabolism, we measured the bioenergetic profiles of SH-SY5Y cells treated with A1254 in the presence of 3 mM pyruvic acid. O_2_ consumption rates (OCR), an indicator of oxidative phosphorylation (OXPHOS) and extracellular acidification rates (ECAR), an indicator of aerobic glycolysis, were analyzed. As shown in Fig 4, A1254 inhibited OXPHOS when compared to control (vehicle), as indicated by decreased basal OCR (see Fig 4A “Basal”). Furthermore, the amount of basal OCR related to ATP production, calculated as the difference between basal and oligomycin-induced OCR, (Fig 4A “ATP-linked”) was inhibited by A1254 treatment as well as the maximal respiratory capacity, calculated as the difference between the FCCP-stimulated OCR and the OCR after the inhibition with antimycin and rotenone (see Fig 4A “Maximal respiration”). Interestingly, pyruvic acid treatment partially reverted A1254-induced inhibition of basal, ATP-linked and maximal respiration OCR, by increasing the mitochondrial respiratory capacity. Moreover, by means of ECAR, we tested whether A1254 could also hamper the activation of the glycolytic pathway. Specifically, both basal and maximal ECAR were significantly inhibited by A1254 when compared to vehicle-treated cells (Fig 4B “basal”, “Maximal”). In addition, the glycolytic capacity, evaluated as the difference between maximal glycolysis and the treatment with glycolytic inhibitor 2-DG, as well as the glycolytic reserve were significantly inhibited by A1254. Again, pyruvic acid was able to restore the above mentioned parameters (Fig 4B “basal”, “maximal”, “glycolytic capacity” and “glycolytic reserve”).

### Effect of pyruvic acid on Aroclor-1254-induced reduction in the activity of mitochondrial protein complexes

**Fig 5:**
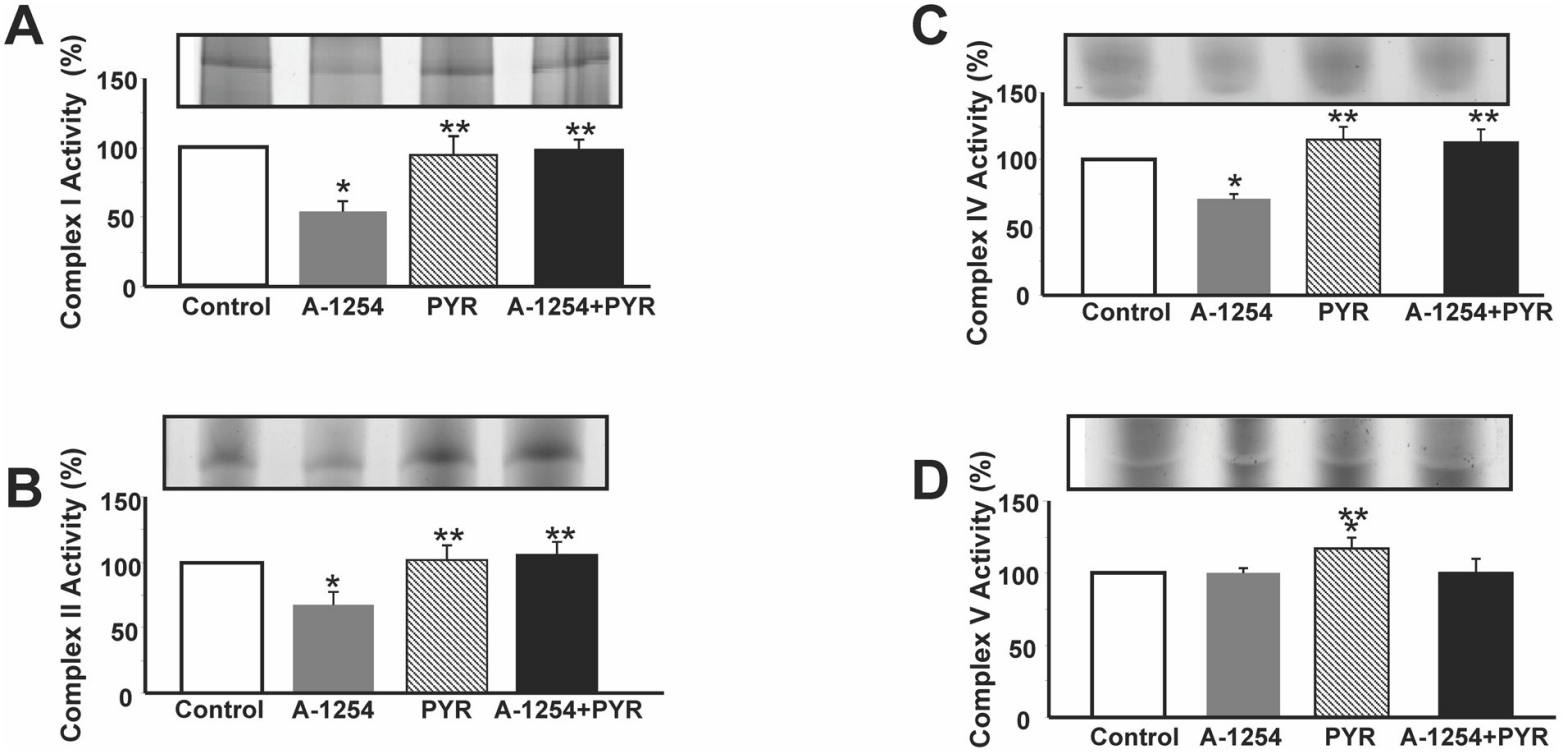
Effect of pyruvic acid on the activity of mitochondrial complexes in SH-SY5Y cells exposed to A1254 evaluated by BN PAGE analysis. Panel A-D. Quantification of gel activity of Complex I, II, IV, and V. Mitochondrial complexes were separated and stained with Coomassie Blue as reported in Method’s section. Data are reported as Δ% of increase vs respective control ± SE; *P<0.05 vs Control group,**P<0.05 vs A1254, ***P<0.05 vs all.

To study the effect of A1254 on the activity of the mitochondrial complexes I, II, IV, and V, these proteins were separated by BN-PAGE and stained with Coomassie Blue, as reported in the methods section. Characteristic bands of individual complexes were already detectable under resting conditions (data not shown). Moreover, to study the specific activity of mitochondrial complexes I,II,IV, and V, mitochondria extracts were prepared from cultured SH-SY5Y cells under resting conditions and after 5 μg/ml Aroclor1254 exposure. A six hour-exposure to A1254 significantly reduced the enzymatic activity of the mitochondrial protein complexes I, II, and IV (Fig 5B, 5C and 5D). Conversely, this treatment did not affect the activity of complex V, that was instead significantly increased by pyruvic acid (Fig 5D). Furthermore, 3 mM pyruvic acid completely restored the activity of mitochondrial complexes I, II and IV (Fig 5A, 5B and 5C).

## Discussion

The present study provides evidence that the exposure to the PCB mixture A1254 causes an increase in intracellular level of nitric oxide, mitochondrial toxicity and cell injury, in human SH-SY5Y neuroblastoma cells. In particular, A1254 reduced mΔΨ, ATP production, mitochondrial complexes I, II, and IV activity and oxidative phosphorylation as well as glycolysis. These effects were observed after a non toxic exposure (6hs) to A1254, whereas a longer exposure (24 hours) led to cell death with an IC50 of ~ 5μg/ml. This evidence suggests that mitochondrial damage underlined A1254-induced cell death in neuronal cells. This is in line with the recent paper by Kodavanti et al. [24] showing the effect of Aroclor 1254 on the alteration of energy metabolism and intracellular signaling-associated proteins involved in brain development. Furthermore, pyruvic acid was able to counteract PCB-induced neurotoxicity by restoring mitochondrial enzymatic activity, glycolysis and oxidative phosphorylation. On the one hand, the negative impact of A1254 exposure on glycolysis and oxidative phosphorylation as well as the reduction in the activity of mitochondrial complex subunits suggests that PCBs impaired ATP production and bioenergetic function thus modifying neuronal response to oxidative stress. On the other hand, pyruvic acid, by counteracting A1254-induced mitochondrial dysfunction, could account for the increased neuronal resistance to oxidative stress. This effect led to a prevention of cell death that passed through a restoration of O_2_ consumption rate, oxidative phosphorylation and aerobic glycolysis. Among the free radicals possibly involved in mitochondrial inhibition, it should be considered that nitric oxide provokes the blockade of the glycolytic pathway, ATP depletion, and cell death in neurons [25]. Interestingly, this gaseous mediator is involved in A1254-induced neurotoxicity [7]. In fact, we have demonstrated that A1254 exposure upregulates the expression of the β-isoform of neuronal Nitric Oxide synthase (nNOS) causing an increase in nitrite production [7]. Accordingly, in the present study we showed that A-1254 induced a rapid and significant increase in intracellular nitric oxide level that was reduced by pyruvic acid. Therefore, we here hypothesize that A1254-induced nitric oxide production could induce mitochondrial injury.

One possible explanation is that nitric oxide can exert a toxic effect on mitochondria by its binding to cytochrome-c-oxidase. Eventually, such binding decreases the mitochondrial affinity for O_2_, thus affecting mitochondrial electron transport and ATP synthesis [11]. Consistently, endogenous formation of nitric oxide by glutamate receptor activation in cortical neurons leads to a rapid and reversible inhibition of mitochondrial ATP synthesis [8]. Another possible mechanism eliciting A1254-induced neurotoxicity is that PCB can stimulate an increase in peroxynitrite production [11, 12, 26]. This evidence is in line with earlier studies demonstrating that peroxynitrite production causes a persistent inhibition of complexes I and IV [9]. One relevant finding emerging from the present study is that A1254 exposure did not affect complex V (i.e. ATP synthase) activity, suggesting that the reduction in ATP levels is not a direct consequence of PCB exposure but it is likely triggered by reduction of the activity of a complex IV, the most sensitive to nitric oxide [11]. Another relevant finding is the neuroprotective effect exerted by pyruvic acid on A1254-induced injury. In fact, this compound was able to prevent SH-SY5Y cell death induced after 24 hrs of A1254 exposure. We can hypothesize that pyruvate-induced neuroprotection is related to an improvement in mitochondrial function. This hypothesis is corroborated by our data showing that pyruvic acid was able to prevent A1254-induced reduction of mΔΨ, the driving force for mitochondrial ATP production. Consistently, pyruvic acid partially restored ATP levels previously dysregulated by A1254 exposure. It is conceivable that pyruvic acid by bolstering the cytosolic energy state [27] together with mitochondrial energy metabolism, prevented neuroblastoma cell death induced by A1254. However, an antioxidant effect of pyruvic acid cannot be ruled out. Indeed, it has been shown that pyruvic acid exerts antioxidant effects in *in vitro* and *in vivo* models of neurodegeneration induced by β-amyloid [13], H2O2 [14,15,18], mitochondrial toxins [28], and zinc [29–30], in which mitochondrial dysfunction play a crucial role in necrotic or apoptotic cell death. Furthermore, pyruvic acid was able to prevent neuronal cell death induced by PCB most likely by acting on death pathways already characterized [31–32]. Collectively, we have shown that a decline in mitochondrial function plays a key role in the chain of events that lead to human neuroblastoma SH-SY5Y cell death after exposure to A1254 and that pyruvic acid can efficiently counteract A1254-induced neurotoxicity by restoring mitochondrial membrane potential, ATP production, the activity of the mitochondrial complexes I, II, and IV and oxidative phosphorylation as well as glycolysis.
